# Compound heterozygous *WNT10A* missense variations exacerbated the tooth agenesis caused by hypohidrotic ectodermal dysplasia

**DOI:** 10.1186/s12903-024-03888-5

**Published:** 2024-01-27

**Authors:** Yiting Liu, Jing Sun, Caiqi Zhang, Yi Wu, Siyuan Ma, Xuechun Li, Xiaoshan Wu, Qingping Gao

**Affiliations:** 1 The Stomatology Center of Xiangya Hospital, Central South University, 87 Xiangya Road, Changsha, Hunan 410008 China; 2 Academician Workstation for Oral & Maxillofacial Regenerative Medicine, Central South University, Changsha, Hunan Province China; 3 Research Center of Oral and Maxillofacial Development and Regeneration, Xiangya Hospital, Central South University, Changsha, Hunan Province China; 4 National Clinical Research Center for Geriatric Diseases, Xiangya Hospital, Central South University, Changsha, Hunan Province China; 5 Beijing Laboratory of Oral Health, Capital Medical University, Beijing, China

**Keywords:** Hypohidrotic ectodermal dysplasia, EDA, WNT10A, Digenic variations, Tooth agenesis, Development

## Abstract

**Background:**

The aim of this study was to analyse the differences in the phenotypes of missing teeth between a pair of brothers with hypohidrotic ectodermal dysplasia (HED) and to investigate the underlying mechanism by comparing the mutated gene loci between the brothers with whole-exome sequencing.

**Methods:**

The clinical data of the patients and their mother were collected, and genomic DNA was extracted from peripheral blood samples. By Whole-exome sequencing filtered for a minor allele frequency (MAF) ≤0.05 non-synonymous single-nucleotide variations and insertions/deletions variations in genes previously associated with tooth agenesis, and variations considered as potentially pathogenic were assessed by SIFT, Polyphen-2, CADD and ACMG. Sanger sequencing was performed to detect gene variations. The secondary and tertiary structures of the mutated proteins were predicted by PsiPred 4.0 and AlphaFold 2.

**Results:**

Both brothers were clinically diagnosed with HED, but the younger brother had more teeth than the elder brother. An *EDA* variation (c.878 T > G) was identified in both brothers. Additionally, compound heterozygous variations of *WNT10A* (c.511C > T and c.637G > A) were identified in the elder brother. Digenic variations in *EDA* (c.878 T > G) and *WNT10A* (c.511C > T and c.637G > A) in the same patient have not been reported previously. The secondary structure of the variant WNT10A protein showed changes in the number and position of α-helices and β-folds compared to the wild-type protein. The tertiary structure of the WNT10A variant and molecular simulation docking showed that the site and direction where WNT10A binds to FZD5 was changed.

**Conclusions:**

Compound heterozygous *WNT10A* missense variations may exacerbate the number of missing teeth in HED caused by *EDA* variation.

**Supplementary Information:**

The online version contains supplementary material available at 10.1186/s12903-024-03888-5.

## Introduction

Tooth agenesis (TA) is divided into nonsyndromic and syndromic types according to the presence or absence of multiple clinical syndromes [1, 2]. Nonsyndromic tooth agenesis (NSTA) is by far the most common form of tooth agenesis [2]. NSTA presents as an isolated trait that only affects dentition. Epidemiological studies indicate that the prevalence of NSTA ranges from 2.6 to 11.3% in various nations and races [3–5]. Numerous genes account for the aetiology of NSTA, *MSX1*, *PAX9*, *AXIN2*, *WNT10A*, *WNT10B*, *LRP6*, *EDA* are most frequently mentioned in relationship to TA [6–12]. *WNT10A*, which is located on chromosome 2q35, has been reported as a major pathogenic gene suggested to account for as much as 50% of NSTA cases [13, 14].

TA can also be part of a complex disorder in a syndromic form, which is known as syndromic tooth agenesis, such as ectodermal dysplasia (EDs). EDs are classified into 11 clinical subgroups, and hypohidrotic ectodermal dysplasia (HED) is one of the most common type of EDs [2, 15, 16], presenting with hypodontia/oligodontia, hypohidrosis/anhidrosis, and hypotrichosis [16–18]. Additional dysmorphic features may be associated with HED, including a prominent forehead, rings under the eyes, saddle nose, prominent protruding lips, periocular pigmentation, alterations in the meibomian glands, and the sporadic absence of nipples [18–20]. Four genes are known to cause about 90% of hypohidrotic/anhidrotic ED cases to date: *EDA*, *EDAR*, *EDARADD*, and *WNT10A* [18]. The majority of HED cases are associated with variations or deletions in *EDA* gene (Xq12-q13.1) inherited on the X-chromosome. The *WNT10A* gene was determined to be responsible for various autosomal recessive forms of EDs, including onycho-odonto-dermal dysplasia (OODD) and Schöpf-Schulz-Passarge syndrome [21, 22]. Moreover, recent studies revealed that *WNT10A* gene variations could also cause HED [18, 22, 23]. Compared to HED cases caused by *EDA* variations, the clinical phenotype of HED caused by *WNT10A* variations is milder, exhibiting abnormal hair and sweat glands but no facial deformities [18]. And the remaining 10% of hypohidrotic/anhidrotic ED cases caused by rarer genetic variations, such as *NFKBIA* [24–26], and *NEMO*/*IKBKG* [27–29]. However, the molecular mechanisms and signalling pathways underlying HED with *WNT10A* variations have not been fully elucidated and the correlation between the number and location of missing teeth in HED and the pathogenic genes has not yet been fully elucidated.

In our clinical study, a pair of brothers were diagnosed with HED, and both had the characteristic features of hypodontia, hypotrichosis, hypohidrosis, and facial dysmorphism. Interestingly, the dental hypoplasia of the elder brother was much more severe than that of the younger brother. The elder brother presented with the mandibular edentulous jaw and only two maxillary central incisors, while the younger brother had some anterior teeth. To study the genetic pathogenesis, whole-exome sequencing (WES) was performed. The results revealed that the elder brother had both *EDA* and *WNT10A* variations, while his younger brother only had the *EDA* variation. To explore whether the mutated *WNT10A* protein had impaired function, we predicted its three-dimensional structure and analysed the functional changes in the mutated protein.

## Materials and methods

### Clinical data collection

Two brothers (11-year-old and 8-year-old) were referred to the Center of Stomatology, Xiangya Hospital, Central South University (China), complaining of congenital missing teeth. With informed consent from the mother and the brothers, we collected the patients’ medical history, took photos, and drew peripheral venous blood samples.

### Whole-exome sequencing (WES) and analysis of sequencing results

DNA extraction and WES of qualified DNA samples were performed by Genergy Bio-Technology (Shanghai) Co., LTD.. According to the WES results, firstly, we removed any variation based on a minor allele frequency (MAF) > 0.05 in any of the three database: 1000 Genomes (1000G; https://www.internationalgenome.org/), Exome Aggregation Consortium (ExAC; http://exac.broadinstitute.org/) or Genome Aggregation Database (gnomAD; https://gnomad.broadinstitute.org/), all of which contain normal healthy individuals from the East Asian population who were similar to the patients [30, 31]. Secondly, we removed synonymous single-nucleotide, and filtered all nonsynonymous single-nucleotide variants and insertions/deletions located in the exon regions (Supplementary Table 1). Then, we filtered variations in genes from the list of 55 genes associated with TA reported in previous studies (Supplementary Table 2). Additionally, based on the scoring criteria of the following tools:(1) SIFT score ≤ 0.05, (2) Polyphen-2 score ≥ 0.909, (3) CADD score > 20, (4) meeting ACMG criteria, we identified potential pathogenicity of reserved variations associated with TA.

### Sanger sequencing

To verify the WES results, the related *EDA* and *WNT10A* fragments were sequenced using Sanger sequencing. Genomic DNA from the brothers was isolated according to the procedure using a the TIANamp Blood DNA Midi Kit (Tiangen, Beijing, China) according to the manufacturer’s procedure. The primers used were specifically designed to detect the variations (Table 1). The coding sequences of the *EDA* and *WNT10A* genes were amplified using PCR with Taq PCR Master Mix (BioTek, Beijing, China). The PCR products were sequenced by Tsingke Biotechnology Co., Ltd. (Beijing). The results were compared with the reference sequences for each gene (*EDA*, NM_001399; *WNT10A*, NM_025216) (UCSC, http://genome.ucsc.edu/) to verify the results of WES.

**Table 1:** Gene variations and primer sequences

Variation	Forward primer	Reverse primer
*EDA* c.878 T > G (p.L293R)	5′-AAGTTTGGCCTTCTAGGCTACC-3′	5′-CCTGCACCGGATCTGCATTC-3′
*WNT10A* c.511C > T (p.R171C)	5′-CGCTTTTGCCTACGCCATC-3′	5′-AACTCGGTTGTTGTGAAGCC-3′
*WNT10A* c.637G > A (p.G213S)	5′-CGCTTTTGCCTACGCCATC-3′	5′-AACTCGGTTGTTGTGAAGCC-3′

### Structure prediction

The National Center for Biotechnology Information (NCBI) (https://www.ncbi.nlm.nih.gov/tools/primer-blast/) was used to query the original amino acid sequences encoded by the disease-related genes. The secondary structures of the wild-type and mutated variants of EDA and WNT10A were predicted using PsiPred 4.0 (http://bioinf. cs.ucl.ac.uk/psipred). Homology modelling analysis and tertiary structure prediction were performed with AlphaFold 2 (https://www.alphafold.ebi.ac.uk/). HDOCK, a web server of molecular docking (http://hdock.phys.hust.edu.cn/), was used to predict of protein-protein interactions. The structures of the wild-type and mutant proteins were generated and compared by PyMOL software (PyMOL Molecular Graphics System; DeLano Scientific).

## Results

### The elder brother has a more severe phenotype of tooth agenesis than the younger brother

**Fig. 1:**
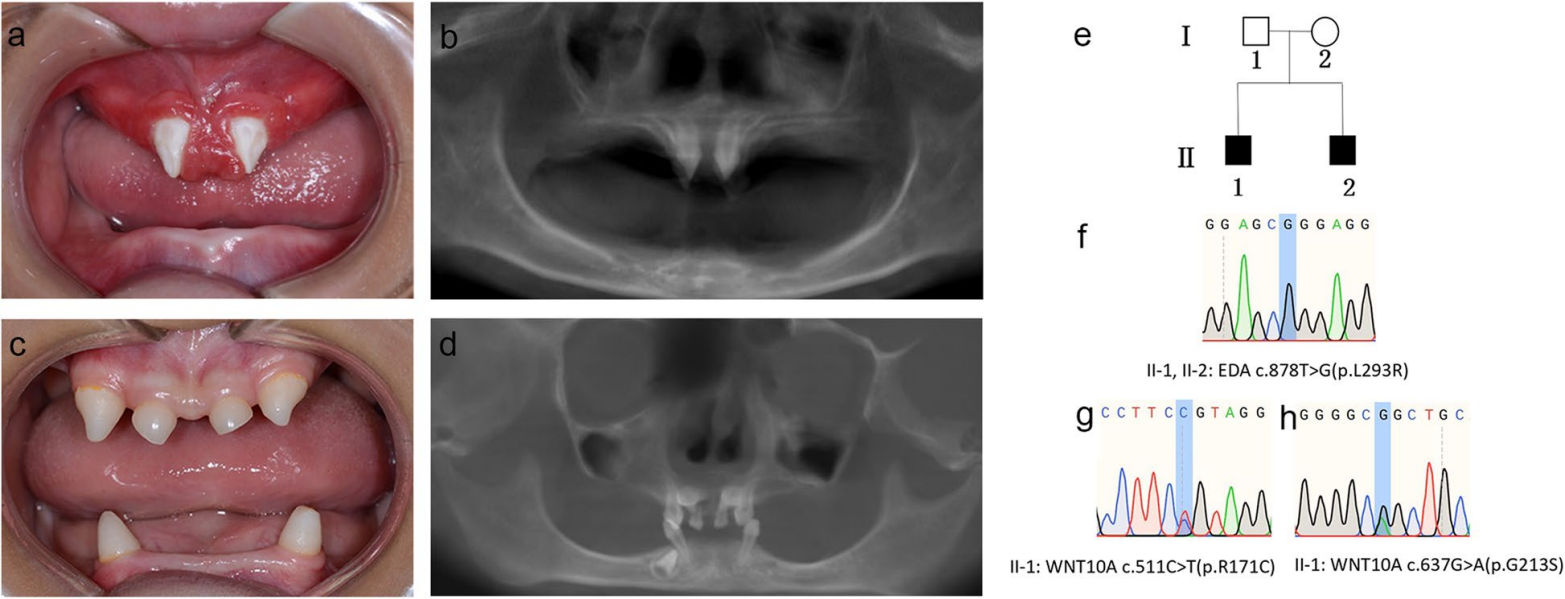
Dental characteristics and facial features of the pair of brothers with HED. **a-b** Oral conditions, panoramic radiographs of the proband (II-1). **c-d** Oral conditions, panoramic radiographs of the little brother (II-2). **e.** Pedigree structure of the family with HED, and black squares represent HED patients. **f.** DNA sequencing chromatogram showing a heterozygous *EDA* variant of c.878 T > G (p.L293R) in the pair of brothers (II-1, II-2). **g-h** Two heterozygous *WNT10A* variants of c.511C > T (p.R171C) and c.637G > A (p.G213S) in the proband (II-1)

Physical examination showed that both brothers presented sparse hair, missing teeth and sweat gland dysplasia (Fig. 1a-d). They both exhibited the typical facial appearance of X-linked HED: saddle nose, prominent thick lips, a pointed chin, and rings under the eyes. Oral examination and cone beam computed tomography (CBCT) examination showed that all primary teeth and most permanent teeth of the elder brother (II-1) were absence congenitally. Only 2 peg-shaped teeth (#11,21) were remained (Fig. 1a-b). Due to the total loss of mandibular dentition, he was unable to chew or construct occlusion. The younger brother (II-2) still had 6 primary teeth (#51,53,61,63,73,83) and 3 permanent teeth germs (#11,21,43) (Fig. 1c-d). Considering the systematic and oral manifestations, both the two brothers were diagnosed with HED.

### A missense variation of *EDA* c.878 T > G (p.L293R) was identified in both brothers, but compound heterozygous *WNT10A* variations (c.511C > T (p.R171C) and c.637G > A (p.G213S)) were found only found in the elder brother

WES screening revealed 3 missense variations in the elder brother, including c.878 T > G (p.L293R) in exon 7 of the *EDA* gene and c.511C > T (p.R171C) and c.637G > A (p.G213S) in exon 3 of the *WNT10A* gene. The three variations were predicted to be damaging by in silico tools (Table 2). The variation sites have been deposited in ClinVar (https://www.ncbi.nlm.nih.gov/clinvar/submitters/509028/). The *WNT10A* variations (c.511C > T and c.637G > A) were compound heterozygous variations identified in the pathogenesis of TA. The younger brother had only the missense variation of c.878 T > G (p.L293R) of *EDA*. Their mother is clinically unaffected and has none of the phenotypes involving hair, sweat glands, or teeth. WES screening revealed that she carries the *EDA* heterozygous variation (c.878 T > G) and one of the *WNT10A* heterozygous variations (c.511C > T). These results were confirmed them by Sanger sequencing (Fig. 1e-h).

**Table 2:** The pathogenicity of three variations were predicted by in silico tools

Variation	dbSNPs	SIFT	PolyPhen-2	CADD	ACMG
*EDA* c.878 T > G (p.L293R)	Unknown	D (0)	D (0.995)	29.1	Uncertain significance PM1 + PM2
*WNT10A* c.511C > T (p.R171C)	rs116998555	D (0)	D (0.93)	32	Uncertain significance PP5 + BS1
*WNT10A* c.637G > A (p.G213S)	rs147680216	D (0.001)	D (0.999)	33	Uncertain significance PP1 + PP3

*SIFT D* Deleterious (≤0.05), *T* Tolerated (> 0.05), *PolyPhen-2 D* Probably damaging (≥0.909), *P* Possibly damaging (0.447 ≤ polyphen-2 ≤ 0.909), *B* Benign (≤0.446). CADD > 20 considered harmful for variation. ACMG PM1: Located in a mutational hot spot and/or critical and well-established functional domain without benign variation; PM2 Absent from controls (or at extremely low frequency if recessive) in Exome Sequencing Project, 1000 Genomes Project, or Exome Aggregation; PP1 Cosegregation with disease in multiple affected family members in a gene definitively known to cause the disease; PP3 Multiple lines of computational evidence support a deleterious effect on the gene or gene product; PP5 Reputable source recently reports variant as pathogenic, but the evidence is not available to the laboratory to perform an independent evaluation; BS1 Allele frequency is greater than expected for disorder

### The mutant protein structures WNT10A and EDA were predicted

**Fig. 2:**
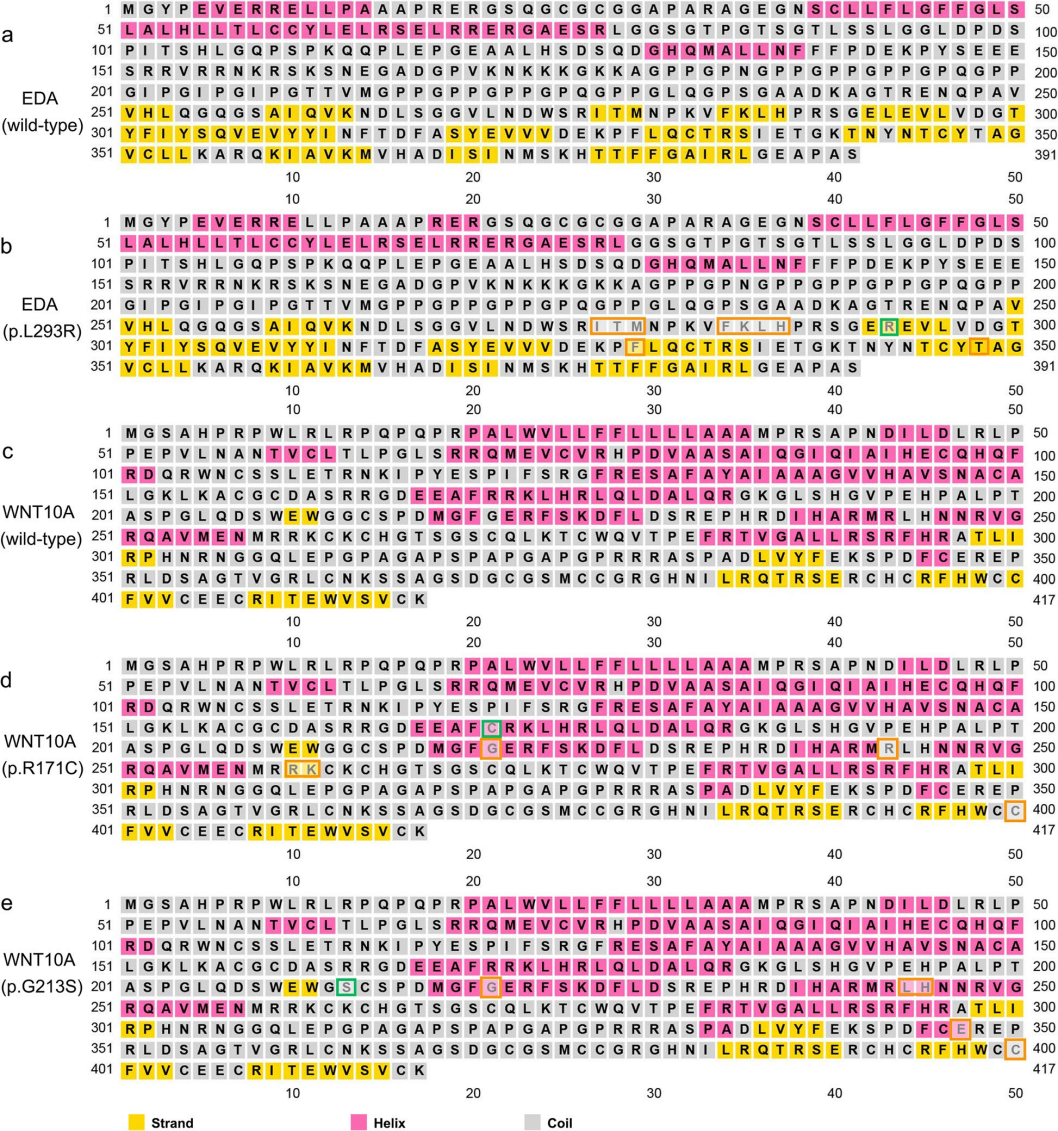
Secondary structure analysis of mutated proteins. **a** The predicted secondary structure of the wild-type EDA protein. **b** The predicted secondary structure of mutated EDA protein (p.L293R). **c.** The predicted secondary structure of the wild-type WNT10A protein. **d.** The predicted secondary structure of mutated WNT10A proteins (p.R171C). **e.** The predicted secondary structure of mutated WNT10A proteins (p.G213S). Sites of variants are indicated by green squares. The structural changes in these mutated proteins compared to the wild-type proteins (EDA and WNT10A) are indicated by orange squares. α-Helices are represented as pink squares, while coils are represented as grey squares

**Fig. 3:**
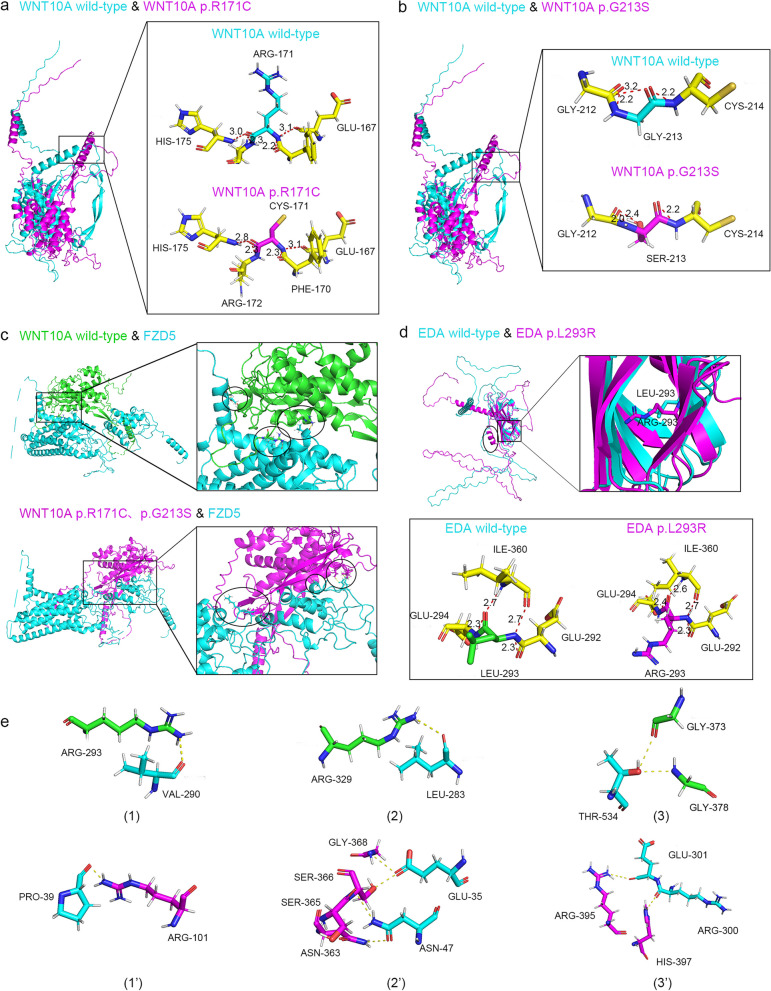
Prediction of tertiary structure of proteins. **a-b** Tertiary structure prediction and hydrogen bond analysis of WNT10A wild-type and WNT10A p.R171C and p.G213S. **d** Tertiary structure prediction and hydrogen bond analysis of EDA wild-type and EDA p.L293R. **c**,** e **(1)–(3). Simulation of molecular docking between WNT10A wild-type and FZD5, and three binding sites between them. **c**,** e **(1′)-(3′). Simulation of molecular docking between WNT10A (p.R171C and p.G213S) and FZD5, and three binding sites between them

The secondary and tertiary protein structures were predicted to analyse the effects of the variants on the protein functions. The secondary structures of the variant EDA (p.L293R) and WNT10A (p.R171C, p.G213S) proteins were predicted and demonstrated that these three missense variants could lead to changes in multiple α-helices and β-folds in the secondary structure of EDA and WNT10A proteins (Fig. 2a-e). Tertiary structure analysis of the EDA (p.L293R) protein showed that the length of the hydrogen bonds between amino acid 293 and amino acid 360 was changed, which results in an extra α-helix of the protein structure (Fig. 3d). In the variant WNT10A (p.R171C, p.G213S) protein, the leucine was replaced by the serine, and the length of the hydrogen bonds formed between the mutated amino acids and the surrounding amino residues was changed (Fig. 3a-b).

FZD5 has been reported to interact with numerous WNT proteins including WNT10A, and to activate signalling cascades in various cellular and developmental processes [32–34]. To explore the possible mechanism of the altered WNT signal, we predicted the ligand-protein molecular docking of the compound heterozygous mutant (p.R171C and p.G213S) protein with FZD5. Compared with ligand-protein molecular docking of the wild-type WNT10A with the FZD5 α-helix, the WNT10A variant was found to mainly bind to the FZD5 loop domain (Fig. 3c). In addition, we revealed that both WNT10A wild-type and WNT10A variant had three binding sites with FZD5 (Fig. 3c, e). The binding sites of the wild-type WNT10A were located at the C-terminal thumb domain of FZD5, while the variant WNT10A not only binds to the C-terminal thumb domain of FZD5, but also to the N-terminal index finger domain of FZD5. The variation changed the position and direction of the binding site, resulting in a changed dimer structure formed by compound heterozygous mutant WNT10A and FZD5. It suggests compound heterozygous WNT10A mutants may have a negative effect on WNT signalling. In addition, we also predicted the binding of WNT10A (p.R171C) and WNT10A (p.G213S) to FZD5, respectively and we found that the dimers WNT10A (p.R171C) -FZD5 and WNT10A (p.G213S)-FZD5 were almost identical in structure to the wild-type WNT10A-FZD5 dimer (Fig. 3c, Supplementary Fig. 1a-b), although their binding sites were not exactly the same (Supplementary Fig. 1c-d).

## Discussion

HED displays different modes of inheritance according to the gene that is involved, with X-linked *EDA*-related HED being the most frequent form of the disease. *EDA* is located in the Xq12-q13 region, which has multiple subtypes and mainly mediates epidermal-mesenchymal and cell–cell signalling transduction [35–37]. Classical EDA signaling components include EDA, EDAR and EDARDD, and the downstream signalling pathway is the NF-κB signalling pathway [38, 39]. The *EDA* gene has 4 important functional areas [40–42]: TM, Furin cleavage, Collagen-like domain (collagen) and TNF homology domain, the latter of which is the most commonly mutated structure [43]. The c.878 T > G variation found in our study [44], is located in the TNF homology domain of the *EDA* gene. The TNF homology domain binds to the receptor (EDAR) to form an autotrimer. We speculate that the c.878 T > G variation in the TNF homology domain prevents EDA from binding to EDAR and therefore affects EDA signal transduction, resulting in increased expression of IκBα that the most important member in inhibiting the activation of NF-κB [45, 46] by inhibiting the phosphorylation of NF-κB, nuclear transcription and binding to DNA, which is downstream of the NF-κB signalling pathway. The inactivation of the NF-κB signalling pathway leads to the developmental defects in ectoderm-derived tissues and organs [47, 48]. Our finding was similar to that of a previous study, in which a patient with the c.878 T > G variation had only two peg-shaped central incisors remaining in the upper jaw [44].

**Fig. 4:**
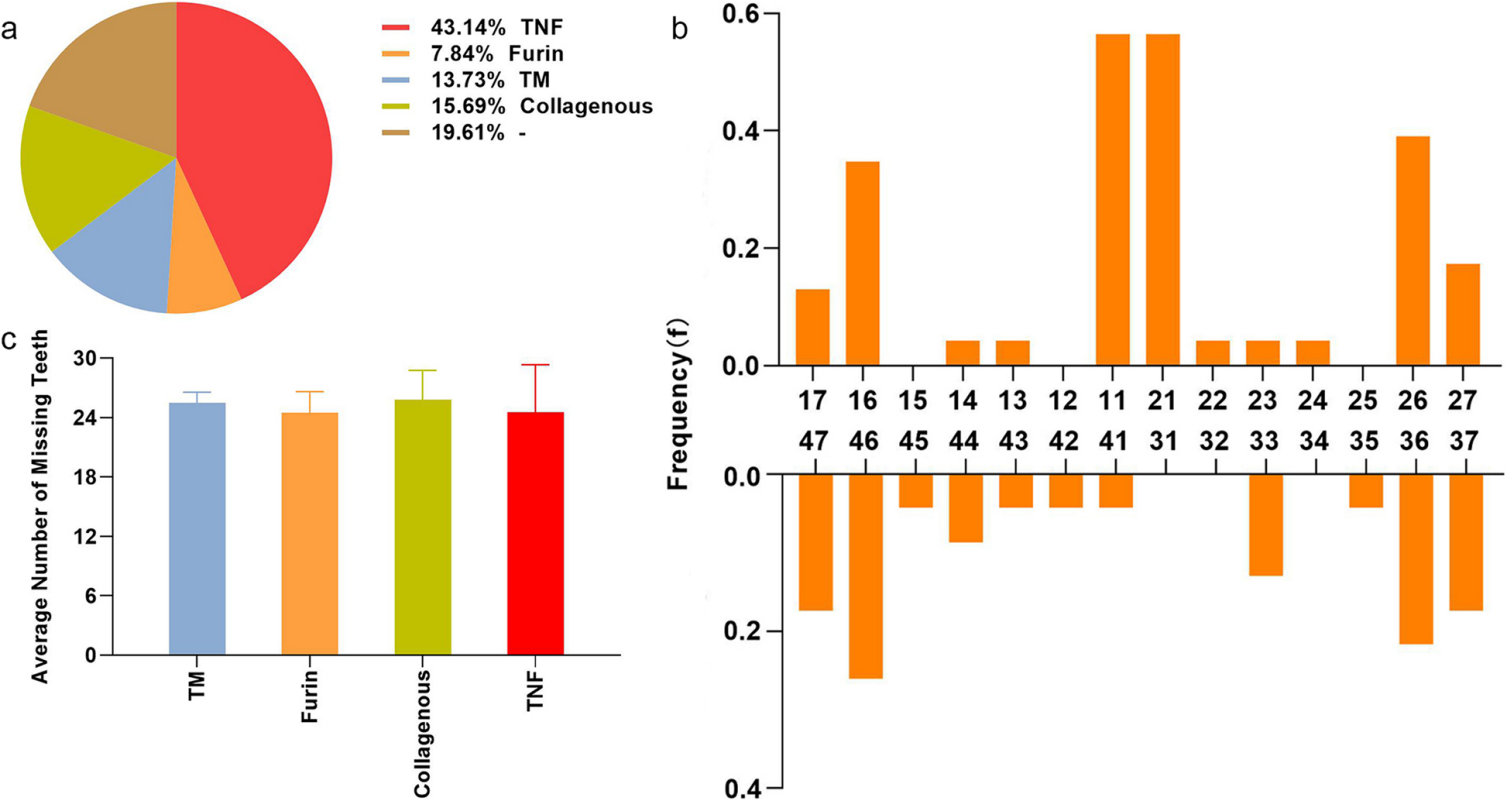
Summary of molecular genetic data of *EDA* variations caused HED. **a** The distribution of various variation domains of *EDA* in HED patients. **b** The frequency of tooth retention caused by EDA variations at each dental position. **c** The average number of missing teeth caused by variations in each functional domain of EDA. There was no significant difference between groups (*P* > 0.5) by Kruskal-Wallis *H* test

Large *EDA* variations identified in HED have been reported, and the phenotypes of these variations vary. In this study, we summarized the HED phenotypes caused by *EDA* variations in the past 5 years (Table 3). We found statistically that 43.14% (22/51) of *EDA* variations was located in the TNF homology subdomain (Fig. 4a), which is consistent with previous reports [43]. The average number of missing teeth caused by variations in different *EDA* domains was greater than 24, with no significant differences (Fig. 4c), which is consistent with the phenotype of the patients in this study. Different tooth positions were affected; the maxillary central incisor was the least affected, with a retention rate of 0.538 (14/26, #11 and #21), followed by left maxillary first molars with a retention rate of 0.385 (10/26, #26) (Fig. 4b). As shown in Table 3, patients with the c.457C > T variation all retained the upper and lower first molars, and patients with the c.164 T > C variation retained the maxillary central incisors. In addition, we found that patients with the same *EDA* variation had differences in missing tooth phenotypes, which may be caused by epigenetics or other variations that have not been detected.

**Table 3:**
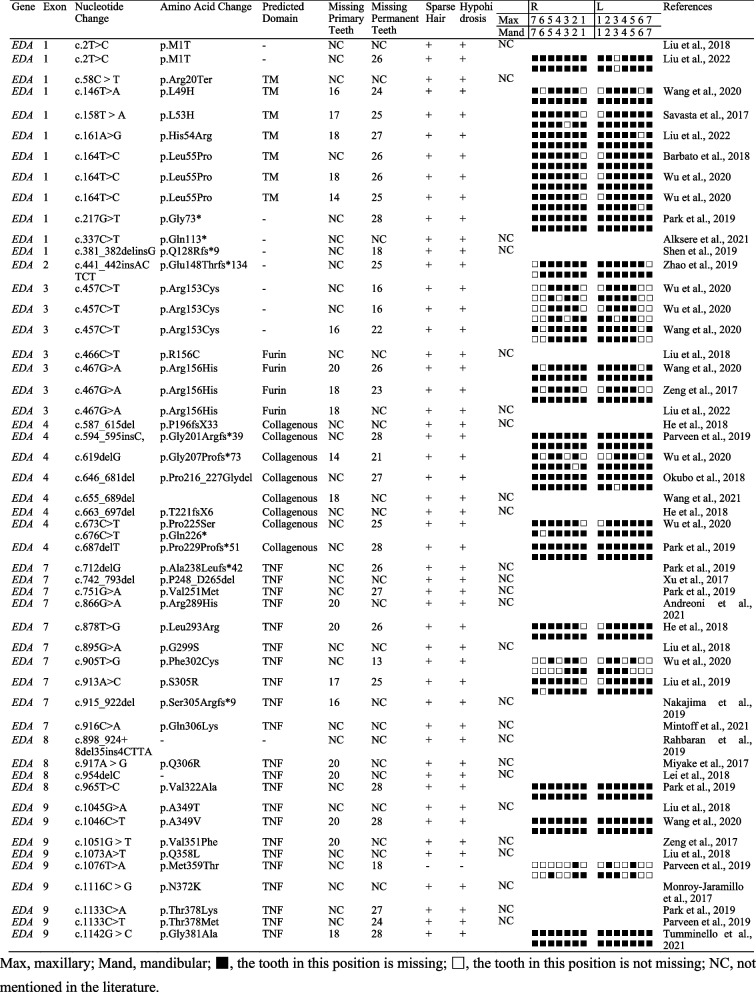
Summary of clinical and molecular genetic data of *EDA* variations caused HED in the past 5 years [44, 49–69]

*WNT10A* is considered to be a common pathogenic gene in NSTA, and is located at chromosome 2q35 [70]. By acting as a short-range ligand, WNT10A locally activates the receptor-mediated WNT signalling pathway [33]. WNT10A was found to be expressed in the dental epithelium and the enamel knot, as well as in the mesenchymal preodontoblast layer during tooth development and is required for dentinogenesis and tooth morphogenesis [71, 72]. WNT10A comprises an N-terminal index finger domain with α-helices (NTD) from residue 1 to residue 250, and a C-terminal cysteine-rich region (CTD) with a thumb from residue 261 to residue 338. *WNT10A* c.511C > T (p.R171C) and c.637G > A (p.G213S), located in exon 3, are the two frequent variations in Asian NSTA population [73, 74]. The compound heterozygous *WNT10A* variations (c.511C > T and c.637G > A) had been reported in a male who diagnosed with NSTA [75]. He was two teeth absent clinically, without any symptoms of ectodermal dysplasia [75]. The two variations are both located in the N-terminal index finger domain, near 4 disulfide bonds [34], and the variations of amino residues may destroy disulfide bond binding, affect the stability of protein structure, and eventually lead to destabilizing of the N-terminal index finger domain [34, 76]. Neither variation has been reported in cases of HED, although the of c.637G > A variation has been reported to cause minor signs of ED [77]. Currently, it is believed that the tooth loss phenotype caused by compound heterozygous *WNT10A* variations is far more serious than that caused by a single heterozygous *WNT10A* variation [78].

In this study, we collected cases of TA with concurrent *EDA* and *WNT10A* digenic variations (Table 4). In a previous study on TA by *WNT10A* and *EDA* digenic variations, patients with simple heterozygous *WNT10A* and *EDA* digenic variations had no more severe phenotypes than those previously reported with *EDA* single variations [78]. In this study, which compared a pair of biological brothers, may have excluded the influence of other confounding factors. We found that the presence of two variations (c.511C > T and c.637G > A) in *WNT10A* led to a more severe absence of teeth in HED patients.

**Table 4:**
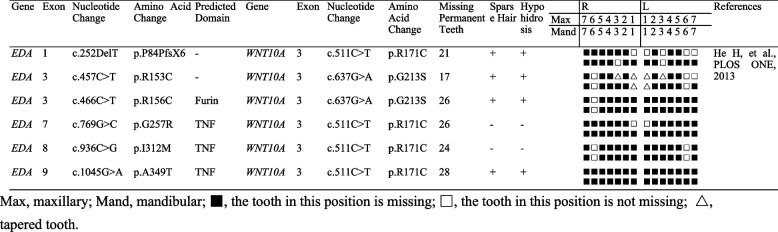
Summary of clinical and molecular genetic data of *EDA* and *WNT10A* digenic variations [78]

The EDA/EDAR/NF-κB signalling pathway is now recognized as a classical signalling pathway associated with the incidence of HED [16, 18]. Studies have shown the EDA/EDAR/NF-κB signalling pathway is closely related to the Wnt/β-catenin signalling pathway, and they regulate each other. During hair follicle development, Wnt/β-catenin signaling is required for NF-κB activation, while EDAR/NF-κB subsequently enhances and maintains Wnt/β-catenin activity [79]. Meanwhile, during tooth morphogenesis, Wnt10A and EDAR are both expressed in the dental epithelium at initiation and bud stages and in the enamel knot in the cap stage [76]. Wnt signals regulate ectodysplasin expression in the oral ectoderm, and EDAR expression in the epithelial signalling centres is responsive to Wnt-induced ectodysplasin from the nearby ectoderm [32]. As an inhibitor of Wnt signalling, Dkk4 is a direct transcriptional target of EDA/EDAR signal during lamina formation [80]. Lef-1 is known to play a role in Wnt signalling and transcriptional activation. Recent studies showed that overexpression of both Lef-1 and β-catenin significantly increased EDA transcription, and indirect stabilization of endogenous β-catenin stimulated EDA transcription [48, 70]. The WNT10A protein is a member of the Wnt ligand family [81], which activates the canonical Wnt/β-catenin signalling pathway [21, 82, 83]. In this study, compared with the younger brother, the elder brother exhibited a clinical phenotype with more missing teeth. We speculate that the additional WNT10A variations decreased the binding of mutant WNT10A to Wnt receptor genes (FZD5), resulting in the simultaneous damage of the NF-κB and Wnt signalling pathways and the failure of tooth morphogenesis.

In conclusion, compound heterozygous *WNT10A* missense variations may exacerbated the number of missing teeth in HED cause by *EDA* variation. Further study is necessary to determine on how WNT10A interacts with EDA during tooth development.

### Supplementary Information

**Additional file 1.**

**Additional file 2.**

**Additional file 3.**

## Data Availability

The datasets analysed during the current study are available in the ClinVar repository (https://www.ncbi.nlm.nih.gov/clinvar/submitters/509028/). The data that support the findings of this study are available from the corresponding author upon reasonable request.
