# Supplementary material for: Compound heterozygous WNT10A missense variations exacerbated the tooth agenesis caused by hypohidrotic ectodermal dysplasia
Source: BMC Oral Health. 2024 Jan 27;24:136. doi: 10.1186/s12903-024-03888-5 (PMC10822191; doi:10.1186/s12903-024-03888-5)
Supplement: Supplementary file 1 — Additional file 1. [file 12903_2024_3888_MOESM1_ESM.pdf]

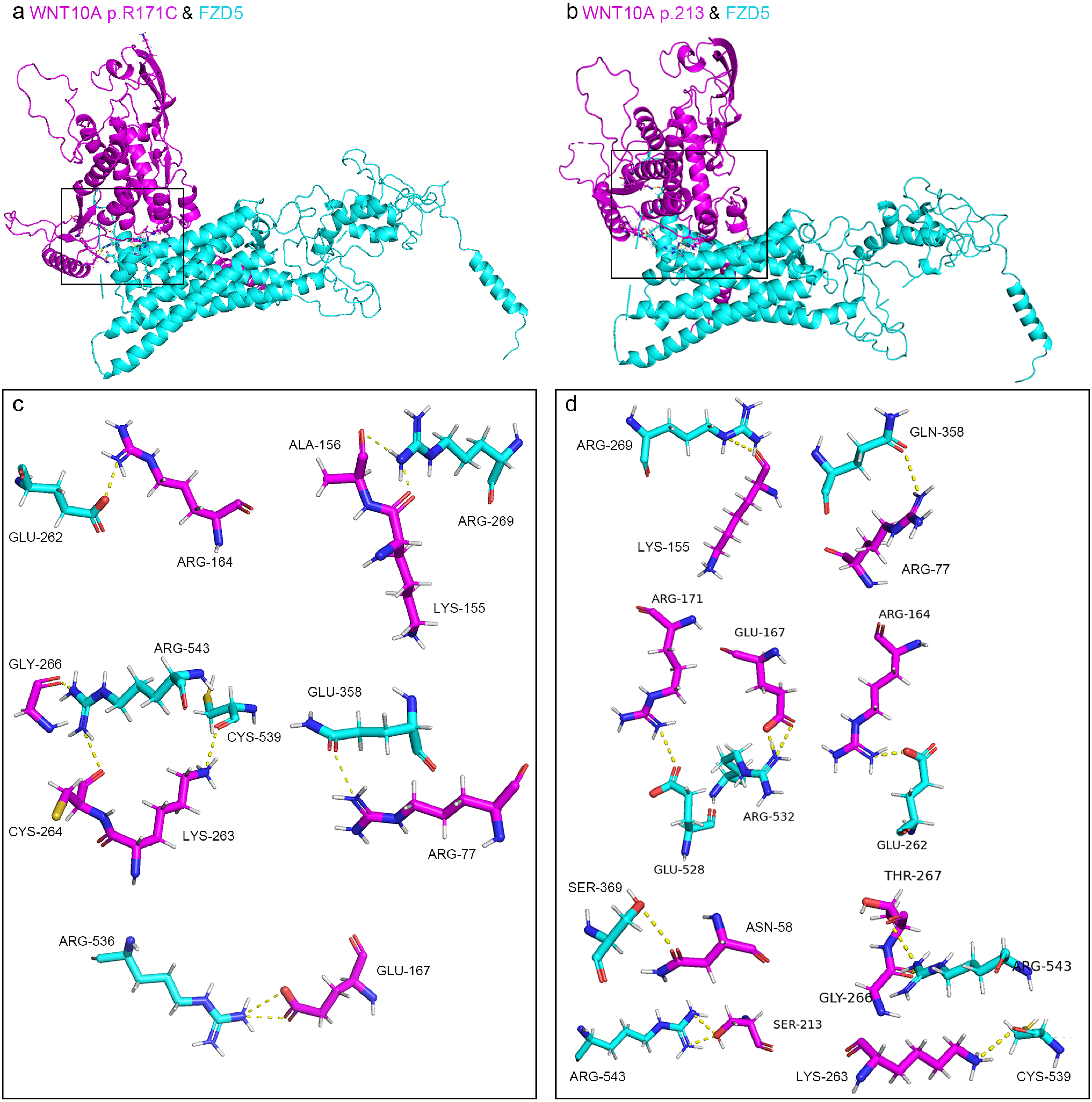

Supplementary Figure 1 Model simulation of the binding of the mutant WNT10A to FZD5. **a.** Simulation of the binding of WNT10A (p.R171C) to FZD5 **b.**Simulation of the binding of WNT10A (p.G213S) to FZD5. **c.** Binding sites between WNT10A (p.R171C) and FZD5. **d.** Binding sites between WNT10A (p.G213S) and FZD5. Mutant WNT10A, FZD5 are colored slate magenta and cyan, respectively. Interactions between proteins are denoted as yellow dashed lines.
