# Supplementary material for: Compound heterozygous WNT10A missense variations exacerbated the tooth agenesis caused by hypohidrotic ectodermal dysplasia
Source: BMC Oral Health. 2024 Jan 27;24:136. doi: 10.1186/s12903-024-03888-5 (PMC10822191; doi:10.1186/s12903-024-03888-5)
Supplement: Supplementary file 3 — Additional file 3. [file 12903_2024_3888_MOESM3_ESM.pdf]

Supplementary Table 2. 55 genes associated with tooth agenesis

Gene symbol(s)	Description	OMIM	References
ANOS1	anosmin 1	300836	[1]
ANTXR1	anthrax toxin receptor 1	606410	[2]
AXIN2	axin 2	604025	[3]
BCOR	BCL6 corepressor	300485	[4]
BMP4	bone morphogenetic protein 4	112262	[5, 6]
CHD7	chromodomain helicase DNA binding protein 7	608892	[7]
CLCN7	chloride voltage-gated channel 7	602727	[8]
DKK1	dickkopf WNT signaling pathway inhibitor 1	605189	[9]
DSP	desmoplakin	125647	[10]
EDA	ectodysplasin A	300451	[11]
EDAR	ectodysplasin A receptor	604095	[12]
EDARADD	ectodysplasin A receptor associated via death domain	606603	[13]
EVC1	EvC ciliary complex subunit 1	604831	[14, 15]
EVC2	EvC ciliary complex subunit 2	607261	[16]
FGFR1	fibroblast growth factor receptor 1	136350	[17]
FGFR2	fibroblast growth factor receptor 2	176943	[18]
FOXC1	forkhead box C1	601090	[19]
GLI2	GLI family zinc finger 2	165230	[20]
GLI3	GLI family zinc finger 3	165240	[20]
GREM2	gremlin 2, DAN family BMP antagonist	608832	[21]
GRHL2	grainyhead like transcription factor 2	608576	[22]
IFT122	intraflagellar transport 122	606045	[23]
IFT43	intraflagellar transport 43	614068	[24]
IKBKG	inhibitor of nuclear factor kappa B kinase regulatory subunit gamma	300248	[25]
IRF6	interferon regulatory factor-6	607199	[26]
KCTD1	potassium channel tetramerization domain containing 1	613420	[27]
KDM6A	lysine demethylase 6A	300128	[28, 29]
KMT2D	lysine methyltransferase 2D	602113	[29]
KREMEN1	kringle containing transmembrane protein 1	609898	[30]
LAMA3	laminin subunit alpha 3	600805	[7]
LAMB3	laminin subunit beta 3	150310	[4]
LRP6	LDL receptor related protein 6	603507	[31]
LTBP3	latent transforming growth factor beta binding protein 3	602090	[32]
MSX1	msh homeobox 1	142983	[33]
NFKBIA	NFKB inhibitor alpha	164008	[34]
PAX9	paired box 9	167416	[35]
PITX2	paired like homeodomain 2	601542	[36]

POLR3A	RNA polymerase III subunit A	614258	[37, 38]
POLR3B	RNA polymerase III subunit B	614366	[38]
PTCH1	patched 1	601309	[39]
SIX3	SIX homeobox 3	603714	[40]
SHH	sonic hedgehog signaling molecule	600725	[40]
SMOC2	SPARC related modular calcium binding 2	607223	[41]
TBCE	tubulin folding cofactor E	604934	[42]
TBX22	T-box transcription factor 22	300307	[43]
TGIF1	transforming growth factor beta induced factor homeobox 1	602630	[40]
TGFB3	transforming growth factor beta 3	190230	[6]
TP63	tumor protein p63	603273	[44]
TRAF6	TNF receptor associated factor 6	602355	[45]
TSPEAR	thrombospondin type laminin G domain and EAR repeats	612920	[4]
UBR1	ubiquitin protein ligase E3 component n-recognin 1	605981	[46]
WDR19	WD repeat domain 19	608151	[24]
WDR35	WD repeat domain 35	613602	[24]
WNT10A	Wnt family member 10A	606268	[47]
WNT10B	Wnt family member 10B	601906	[48]

References

1. Meczekalski B, Podfigurna-Stopa A, Smolarczyk R, Katulski K, Genazzani AR. Kallmann syndrome in women: from genes to diagnosis and treatment. Gynecological Endocrinology 2013, 29(4):296-300.
2. Dinckan N, Du RQ, Akdemir ZC, Bayram Y, Jhangiani SN, Doddapaneni H, Hu JH, Muzny DM, Guven Y, Aktoren O *et al*. A biallelic ANTXR1 variant expands the anthrax toxin receptor associated phenotype to tooth agenesis. American Journal of Medical Genetics Part A 2018, 176(4):1015-1022.
3. Mostowska A, Biedziak B, Jagodzinski PP. Axis inhibition protein 2 (AXIN2) polymorphisms may be a risk factor for selective tooth agenesis. Journal of Human Genetics 2006, 51(3):262-266.
4. Du RQ, Dinckan N, Song XF, Coban-Akdemir Z, Jhangiani SN, Guven Y, Aktoren O, Kayserili H, Petty LE, Muzny DM *et al*. Identification of likely pathogenic and known variants in *TSPEAR*, *LAMB3*, *BCOR*, and *WNT10A* in four Turkish families with tooth agenesis. Human Genetics 2018, 137(9):689-703.
5. Yu M, Wang H, Fan ZZ, Xie CC, Liu HC, Liu Y, Han D, Wong SW, Feng HL. BMP4 mutations in tooth agenesis and low bone mass. Archives of Oral Biology 2019, 103:40-46.
6. Antunes LD, Küchler EC, Tannure PN, Lotsch PF, Costa MD, Gouvêa CVD, Olej B, Granjeiro JM. *TGFB3* and *BMP4* polymorphism are associated with isolated tooth agenesis. Acta Odontologica Scandinavica 2012, 70(3):202-206.
7. Biedziak B, Firlej E, Dabrowska J, Bogdanowicz A, Zadurska M, Mostowska A. Novel Candidate Genes for Non-Syndromic Tooth Agenesis Identified Using Targeted Next-Generation Sequencing. Journal of Clinical Medicine 2022, 11(20):6089.
8. Zhang YL, Ji DR, Li L, Yang SQ, Zhang HW, Duan XH. ClC-7 Regulates the Pattern and Early Development of Craniofacial Bone and Tooth. Theranostics 2019, 9(5):1387-1400.

9. Liu HC, Zhang J, Wong S, Han D, Zhao HS, Feng HL. Association between rs11001553 of DKK1 and non-syndromic tooth agenesis in the Chinese Han population. Genetics and Molecular Research 2014, 13(3):7133-7139.
10. Chalabreysse L, Senni F, Bruyère P, Aime B, Ollagnier C, Bozio A, Bouvagnet P. A New Hypo/Oligodontia Syndrome: Carvajal/Naxos Syndrome Secondary to Desmoplakin-dominant Mutations. Journal of Dental Research 2011, 90(1):58-64.
11. Kere J, Srivastava AK, Montonen O, Zonana J, Thomas N, Ferguson B, Munoz F, Morgan D, Clarke A, Baybayan P *et al*. X-linked anhidrotic (hypohidrotic) ectodermal dysplasia is caused by mutation in a novel transmembrane protein. Nature genetics 1996, 13(4):409-416.
12. Chassaing N, Bourthoumieu S, Cosse M, Calvas P, Vincent MC. Mutations in *EDAR* account for one-quarter of non-*ED1*-related hypohidrotic ectodermal dysplasia. Human Mutation 2006, 27(3):255-259.
13. Baala L, El-Kerch F, Hadj-Rabia S, Munnich A, Lyonnet S, Sefiani A, Smahi A. Autosomal dominant and recessive anhidrotic ectodermal dysplasia are allelic diseases at the EDARRAD locus. American Journal of Human Genetics 2003, 73(5):178-178.
14. Baujat G, Le Merrer M. Ellis-van Creveld syndrome. Orphanet Journal of Rare Diseases 2007, 2:27.
15. Ruiz-Perez VL, Ide SE, Strom TM, Lorenz B, Wilson D, Woods K, King L, Francomano C, Freisinger P, Spranger S *et al*. Mutations in a new gene in Ellis-van Creveld syndrome and Weyers acrodental dysostosis. Nature Genetics 2000, 24(3):283-286.
16. Zhang Z, Bao K, He JW, Fu WZ, Zhang CQ, Zhang ZL. Identification of one novel mutation in the EVC2 gene in a Chinese family with Ellis-van Creveld syndrome. Gene 2012, 511(2):380-382.
17. Costa-Barbosa FA, Balasubramanian R, Keefe KW, Shaw ND, Al-Tassan N, Plummer L, Dwyer AA, Buck CL, Choi JH, Seminara SB *et al*. Prioritizing Genetic Testing in Patients With Kallmann Syndrome Using Clinical Phenotypes. Journal of Clinical Endocrinology & Metabolism 2013, 98(5):E943-E953.
18. Küchler EC, Lips A, Tannure PN, Ho B, Costa MC, Granjeiro JM, Vieira AR. Tooth Agenesis Association with Self-reported Family History of Cancer. Journal of Dental Research 2013, 92(2):149-155.
19. Ito YA, Footz TK, Murphy TC, Courtens W, Walter MA. Analyses of a novel L130F missense mutation in FOXC1. Archives of Ophthalmology 2007, 125(1):128-135.
20. Marañón-Vásquez GA, Dantas B, Kirschneck C, Arid J, Cunha A, Ramos AGD, Omori MA, Rodrigues AS, Teixeira EC, Levy SC *et al*. Tooth agenesis-related GLI2 and GLI3 genes may contribute to craniofacial skeletal morphology in humans. Archives of Oral Biology 2019, 103:12-18.
21. Mostowska A, Biedziak B, Zadurska M, Bogdanowicz A, Olszewska A, Cieslinska K, Firlej E, Jagodzinski PP. *GREM2* nucleotide variants and the risk of tooth agenesis. Oral Diseases 2018, 24(4):591-599.
22. Petrof G, Nanda A, Howden J, Takeichi T, McMillan JR, Aristodemou S, Ozoemena L, Liu L, South AP, Pourreyron C *et al*. Mutations in GRHL2 Result in an Autosomal-Recessive Ectodermal Dysplasia Syndrome. American Journal of Human Genetics 2014, 95(3):308-314.
23. Alazami AM, Seidahmed MZ, Alzahrani F, Mohammed AO, Alkuraya FS. Novel IFT122 mutation associated with impaired ciliogenesis and cranioectodermal dysplasia. Molecular genetics & genomic medicine 2014, 2(2):103-106.
24. Tan W LA, Keppler-Noreuil K. Cranioectodermal Dysplasia. 2013. [https://www.ncbi.nlm.nih.gov/books/NBK154653/].
25. Zonana J, Elder ME, Schneider LC, Orlow SJ, Moss C, Golabi M, Shapira SK, Farndon PA, Wara

DW, Emmal SA *et al*. A novel X-linked disorder of immune deficiency and hypohidrotic ectodermal dysplasia is allelic to incontinentia pigmenti and due to mutations in IKK-gamma (NEMO). American Journal of Human Genetics 2000, 67(6):1555-1562.
26. Vieira AR, Modesto A, Meira R, Barbosa ARS, Lidral AC, Murray JC. Interferon regulatory factor 6 (IRF6) and fibroblast growth factor receptor 1 (FGFR1) contribute to human tooth agenesis. American Journal of Medical Genetics Part A 2007, 143A(6):538-545.
27. Chen XL, Xiang Y, Yang L, Lin Y. The phenotypic characteristics of patients with athelia and tooth agenesis. Annals of Translational Medicine 2021, 9(20):1583.
28. Rihani FB, Altayeh MM, Al-Kilani RZ, Alrejjal RA. Solitary median maxillary central incisor in Kabuki syndrome 2 with novel missense mutation of KDM6A and ABCC8 genes. Journal of Clinical Pediatric Dentistry 2022, 47(2):108-116.
29. Porntaveetus T, Abid MF, Theerapanon T, Srichomthong C, Ohazama A, Kawasaki K, Kawasaki M, Suphapeetiporn K, Sharpe PT, Shotelersuk V. Expanding the Oro-Dental and Mutational Spectra of Kabuki Syndrome and Expression of KMT2D and KDM6A in Human Tooth Germs. International Journal of Biological Sciences 2018, 14(4):381-389.
30. Lee Y, Zhang H, Seymen F, Koruyucu M, Kasimoglu Y, Lee ZH, Hu JCC, Simmer JP, Kim JW. Novel homozygous *KREMEN1* mutation causes ectodermal dysplasia. Oral Diseases 2022, 28(3):843-845.
31. Massink MPG, Creton MA, Spanevello F, Fennis WMM, Cune MS, Savelberg SMC, Nijman IJ, Maurice MM, van den Boogaard MJH, van Haaften G. Loss-of-Function Mutations in the WNT Co-receptor LRP6 Cause Autosomal-Dominant Oligodontia. American Journal of Human Genetics 2015, 97(4):621-626.
32. Dugan SL, Temme RT, Olson RA, Mikhailov A, Law R, Mahmood H, Noor A, Vincent JB. New recessive truncating mutation in *LTBP3* in a family with oligodontia, short stature, and mitral valve prolapse. American Journal of Medical Genetics Part A 2015, 167(6):1396-1399.
33. Vastardis H, Karimbux N, Guthua SW, Seidman JG, Seidman CE. A human MSX1 homeodomain missense mutation causes selective tooth agenesis. Nature genetics 1996, 13(4):417-421.
34. Courtois G, Smahi A, Reichenbach J, Doffinger R, Cancrini C, Bonnet M, Puel A, Chable-Bessia C, Yamaoka S, Feinberg J *et al*. A hypermorphic I kappa B alpha mutation is associated with autosomal dominant anhidrotic ectodermal dysplasia and T cell immunodeficiency. Journal of Clinical Investigation 2003, 112(7):1108-1115.
35. Stockton DW, Das P, Goldenberg M, D'Souza RN, Patel PI. Mutation of PAX9 is associated with oligodontia. Nature Genetics 2000, 24(1):18-19.
36. Fan ZZ, Sun SC, Liu HC, Yu M, Liu ZY, Wong SW, Liu Y, Han D, Feng HL. Novel *PITX2* mutations identified in Axenfeld-Rieger syndrome and the pattern of *PITX2*-related tooth agenesis. Oral Diseases 2019, 25(8):2010-2019.
37. Tewari VV, Mehta R, Sreedhar CM, Tewari K, Mohammad A, Gupta N, Gulati S, Kabra M. A novel homozygous mutation in POLR3A gene causing 4H syndrome: a case report. Bmc Pediatrics 2018, 18:126.
38. Saitsu H, Osaka H, Sasaki M, Takanashi J, Hamada K, Yamashita A, Shibayama H, Shiina M, Kondo Y, Nishiyama K *et al*. Mutations in *POLR3A* and *POLR3B* Encoding RNA Polymerase III Subunits Cause an Autosomal-Recessive Hypomyelinating Leukoencephalopathy. American Journal of Human Genetics 2011, 89(5):644-651.
39. Murata Y, Kurosaka H, Ohata Y, Aikawa T, Takahata S, Fujii K, Miyashita T, Morita C, Inubushi T, Kubota T *et al*. A novel *PTCH1* mutation in basal cell nevus syndrome with rare craniofacial features.

Human Genome Variation 2019, 6:16.
40. Poelmans S, Kawamoto T, Cristofoli F, Politis C, Vermeesch J, Bailleul-Forestier I, Hens G, Devriendt K, Verdonck A, Carels C. Genotypic and phenotypic variation in six patients with solitary median maxillary central incisor syndrome. American Journal of Medical Genetics Part A 2015, 167(10):2451-2458.
41. Ruan WY, Duan XH. A new *SMOC2* mutation within selective tooth agenesis, malformed teeth and dentin dysplasia. Clinical Genetics 2022, 102(4):352-354.
42. Moussaid Y, Griffiths D, Richard B, Dieux A, Lemerrer M, Léger J, Lacombe D, Bailleul-Forestier I. Oral manifestations of patients with Kenny-Caffey Syndrome. European Journal of Medical Genetics 2012, 55(8-9):441-445.
43. Kantaputra PN, Paramee M, Kaewkhampa A, Hoshino A, Lees M, McEntagart M, Masrour N, Moore GE, Pauws E, Stanier P. Cleft Lip with Cleft Palate, Ankyloglossia, and Hypodontia are Associated with *TBX22* Mutations. Journal of Dental Research 2011, 90(4):450-455.
44. Jin JY, Zeng L, Li K, He JQ, Pang XY, Huang H, Xiang R, Tang JY. A novel mutation (c.1010G>T; p.R337L) in TP63 as a cause of split-hand/foot malformation with hypodontia. Journal of Gene Medicine 2019, 21(10):e3122.
45. Fujikawa H, Farooq M, Fujimoto A, Ito M, Shimomura Y. Functional studies for the *TRAF6* mutation associated with hypohidrotic ectodermal dysplasia. British Journal of Dermatology 2013, 168(3):629-633.
46. Sukalo M, Schäflein E, Schanze I, Everman DB, Rezaei N, Argente J, Lorda-Sanchez I, Deshpande C, Takahashi T, Kleger A *et al*. Expanding the mutational spectrum in Johanson-Blizzard syndrome: identification of whole exon deletions and duplications in the *UBR1* gene by multiplex ligation-dependent probe amplification analysis. Molecular Genetics & Genomic Medicine 2017, 5(6):774-780.
47. Kantaputra P, Sripathomsawat W. WNT10A and Isolated Hypodontia. American Journal of Medical Genetics Part A 2011, 155A(5):1119-1122.
48. Yu P, Yang WL, Han D, Wang X, Guo S, Li JC, Li F, Zhang XX, Wong SW, Bai BJ *et al*. Mutations in WNT10B Are Identified in Individuals with Oligodontia. American Journal of Human Genetics 2016, 99(1):195-201.
